# Novel Inhibitor Design for Hemagglutinin against H1N1 Influenza Virus by Core Hopping Method

**DOI:** 10.1371/journal.pone.0028111

**Published:** 2011-11-30

**Authors:** Xiao-Bo Li, Shu-Qing Wang, Wei-Ren Xu, Run-Ling Wang, Kuo-Chen Chou

**Affiliations:** 1 Tianjin Key Laboratory on Technologies Enabling Development of Clinical Therapeutics and Diagnostics (Theranostics), School of Pharmacy, Tianjin Medical University, Tianjin, China; 2 Gordon Life Science Institute, San Diego, California, United States of America; 3 Tianjin Institute of Pharmaceutical Research (TIPR), Tianjin, China; University of Delhi, India

## Abstract

The worldwide spread of H1N1 avian influenza and the increasing reports about its resistance to the current drugs have made a high priority for developing new anti-influenza drugs. Owing to its unique function in assisting viruses to bind the cellular surface, a key step for them to subsequently penetrate into the infected cell, hemagglutinin (HA) has become one of the main targets for drug design against influenza virus. To develop potent HA inhibitors, the ZINC fragment database was searched for finding the optimal compound with the core hopping technique. As a result, the Neo6 compound was obtained. It has been shown through the subsequent molecular docking studies and molecular dynamic simulations that Neo6 not only assumes more favorable conformation at the binding pocket of HA but also has stronger binding interaction with its receptor. Accordingly, Neo6 may become a promising candidate for developing new and more powerful drugs for treating influenza. Or at the very least, the findings reported here may provide useful insights to stimulate new strategy in this area.

## Introduction

In recent years, severe flu-like human cases were reported around the world and subsequently the causative virus was identified as the influenza A virus [1], [2]. The virus was spreading rapidly around the world and had been identified as a new reassortant with three genetic lineages, mainly with a swine origin. Therefore, it was called swine-origin influenza virus (S-OIV). Owing to its extremely rapid human-to-human transmission rate, within only two months the 2009 S-OIV had been detected throughout the entire world. On June 11th, 2009 the World Health Organization (WHO) declared an official pandemic, the first pandemic in the 21st century [3].

Influenza A virus that belongs to the Orthomyxoviridae family is a negative-strand segmented RNA virus, in which the surface membrane proteins are constituted by three important components: M2 proton channel, hemagglutinin (HA), and neuraminidase (NA). The M2 proton channel is responsible for proton conductance vitally important to viral replication. HA is responsible for binding to the surface of the infected cell as a trimer leading to the attachment and subsequent penetration by viruses into the target cell. NA is responsible for cleaving the terminal sialic acid moieties from the receptors to facilitate the elution of the progeny virions from the infected cell [4]. Therefore, any of the three components can be the target for drug design against influenza virus. Recently, stimulated by the successful determination of its high-resolution three-dimensional structure [5], many discussions about the M2 channel have been made in this regard [5], [6], [7], [8], [9]. The two existing M2 drugs, amantadine (Symmetrel) [10] and rimantadine (Flumadine) [10] approved by FDA, are no longer effective because of their inefficacies to influenza virus.

Sialic acid (SA) as a natural ligand combines with both of the glycoproteins (HA and NA) and located at the membrane of host cell, which is the basis of heme-agglutination when viruses are mixed with blood cells and entry of the virus into cells of the upper respiratory tract [11], [12]. According to the mutagenic analysis the residues of both HA1 and NA binding sites are quite conserved for most influenza A strains [13], [14].

Owing to its deep active site cleft, the NA has been an attractive target for drug design. Both zanamivir and oseltamivir were designed by modifying the sialic acid (SA) structure. The two FDA-approved clinical drugs were once successfully used to inhibit the spread of influenza viral progeny [15] by binding to viral surface glycoprotein of neuraminidase (NA) [15]. However, it has also been found from several clinical cases [16], [17], [18] that oseltamivir failed to treat avian influenza virus. It is both antigenic drift (sequence base mutations) and antigenic shift (genetic recombination) of segmented RNA genome of influenza viruses that have caused the NA inhibitor being resistant [19], [20].

HA facilitates viral entry through binding to the host surface sialic acid residues [21]. Accordingly, if HA is blocked at its sialic acid binding site by a small molecule, the viral entry process will be stopped and the penetration of viruses into host cell prevented. In comparison with NA inhibitors, the HA inhibitors were usually more effective in inhibiting influenza virus. For all the HA subtypes (H1-H16) so far identified [22], the HA1 subtype from the recent pandemic H1N1/09 virus was taken as the target for constituent screening and drug design [23].

Despite of many year scientific research efforts, so far there is no clinical available inhibitor against HA1. On the other hand, many studies have indicated that computational approaches, such as structural bioinformatics [24], [25], molecular docking [26], [27], pharmacophore modeling [28], identification of proteases and their types [29], and HIV protease cleavage site prediction [30], [31], can timely provide very useful information and insights for drug development. Encouraged by the aforementioned studies, the present study was initiated in an attempt to find a new anti-influenza compound by screening the fragment database for the optimal constituent inhibitor. Meanwhile, the techniques of the core hopping with glide docking and molecular dynamic simulation were also utilized to analyze the binding interactions between the inhibitor and HA1, in hopes that the findings thus obtained will be useful for developing new and powerful drugs against H1N1 influenza virus.

## Materials and Methods

### 1. Protein structure and the databases

**Figure 1:**
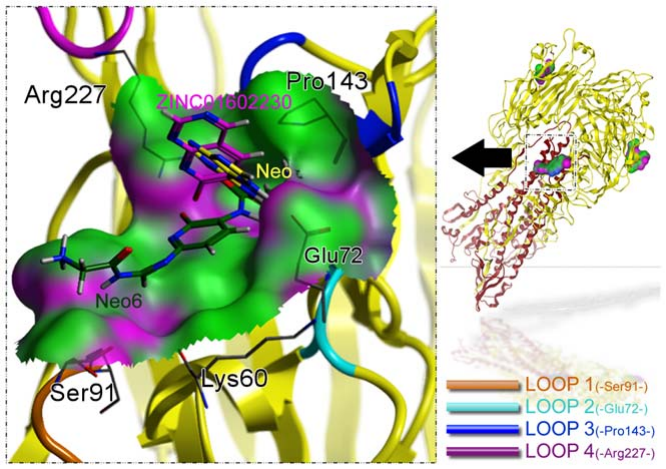
A close-up view for the binding site of HA1 (PDB ID: 3AL4). The binding pocket is defined by those residues that have at least one heavy atom with a distance 5 Å from the NAG ligand [37]. The four loops (loop1, loop2, loop3, and loop4) that play an important role in interacting with the ligand are represented by round ribbons of four different colors as well as their key residues Ser92, Glu72, Pro143, and Arg227, respectively. The motions of such four residues were monitored during the molecular dynamic simulation. The docked poses for ZINC01602230, Neo and Neo6 are shown with the stick model colored in purple, yellow and dark green, respectively.

The crystal structure for the HA1 subtype from the recent pandemic H1N1/09 virus was downloaded from the PDB Bank [32]. Its PDB ID is 3AL4. The antigenicity of the HA1 from the swine-origin A (H1N1)-2009 influenza A virus is quite similar to that of the HA from the 1918 pandemic virus [23]. The binding-site was identified by the SiteMap tool in Schrodinger Suite 2009 (www.schrodinger.com) as described in [34], [35], [36]. The bind-site encompassed the ligand N-Acetyl-D-Glucosamine (NAG), which is observed in complex with HA1 of the crystal structure (PDB: 3AL4). Shown in **Fig. 1** is a close-up view for the binding site of protein HA1 rendered by the molecular surface. The binding pocket is formed by those residues that have at least one heavy atom (i.e., an atom other than hydrogen) with a distance 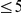 Å away from any heavy atom of NAG ligand when it is bound to the receptor at the binding site, as elaborated in [37]. The segments of loop1, loop2, loop3, and loop4, which play an important role in the interactions with the ligand, are shown by four different colors with their respective key residues: Ser92, Glu72, Pro143, and Arg227 (**Fig. 1**). The motions of such four residues were monitored during the molecular dynamics simulations.

The drug-like database and the fragment database derived from ZINC [38] were used for virtual screening and core hopping searching, respectively.

### 2. Virtual screening

The Glide5 docking program [39] interfaced with Schrodinger Suite 2009 [33] was used to screen the drug-like database from ZINC [38] based on the 3D structure of 3AL4. The preparation and refinement protocols for protein receptor and all compound structures were performed on the Protein Preparation Wizard and LigPrep modules embedded in Schrodinger 2009 [33], respectively. For protein preparation, the process included assigning bond orders, adding hydrogen, treating metals, treating disulfides, deleting waters and alleviating potential steric clashes, adjusting bond order and formal charges by protein minimization with the OPLS2005 force field [40], the constrained refinement value of RMSD for the protein was limited to 0.3 Å. Meanwhile, for the compounds, the preparation consisted of the generating possible states by ionization at target pH 7.0±2.0, desalting, retaining chiralities from 3D structure and geometry minimization with the OPLS2005 force field [40]. When the above steps were accomplished, all investigated compounds were docked into the receptor pocket through the rigid docking model with the Stand-precision (SP) scoring function [41], [42] to estimate the binding affinities.

### 3. Core hopping method

Many useful clues for drug design can be achieved through molecular docking studies (see, e.g., [24], [27], [43], [44], [45], [46]). In order to gain even more useful information in this regard, the novel drug design algorithm called “Core Hopping” [33] was used in this study that has the function to perform both the fragment-based replacing and molecular docking. Such method is particularly useful for de novel drug design because it can improve the activity of the template, which was ZINC01602230 compound in this study. As a lead compound screened out from the drug-like database, the template was taken as an initial structure to subject to optimization via the core hopping method by finding the optimal cores that are attached to the scaffold part of the template in binding with the protein receptor.

During the process of core hopping, the first step was to define the points at which the cores were attached to the scaffold. It was performed in the Define Combinations Step from the Combinatorial Screening panel [33]. The second step was to define receptor grid file, which was done in the Receptor Preparation panel [33]. The third step was to prepare the cores attached to the scaffold for the fragment database derived from ZINC [38]. Finally, the cores thus obtained were sorted and filtered by goodness of alignment and then re-docked into the receptor after attaching the scaffold, followed by using the docking scores to sort the final molecules.

### 4. Molecular dynamics simulations

Many marvelous biological functions in proteins and DNA and their profound dynamic mechanisms, such as switch between active and inactive states [47], [48], cooperative effects [49], allosteric transition [50], [51], intercalation of drugs into DNA [52], and assembly of microtubules [53], can be revealed by studying their internal motions [54]. Likewise, to really understand the action mechanism of a receptor with its ligand, we should consider not only the static structures concerned but also the dynamical information obtained by simulating their internal motions or dynamic process.

In order to examine whether the designed inhibitor remains bound in the presence of explicit solvent from a dynamic point of view, the molecular dynamic simulation was performed with GROMACS 96-53a6 force fields [55] with the periodic boundary conditions (PBC) by using GROMACS 4.0 package for Linux. The topology files and charges for the ligand atoms were generated by the Dundee PRODRG2.5 Server (beta) [56]. Before starting the simulations, all the models were solvated with the explicit simple point charge (SPC) water in a cubic box. The models were covered with a water shell of 1.0 nm from the surface of the protein. The system was neutralized with six chlorine ions to replace the six SPC water molecules. Subsequently, the energy minimization was performed for the system concerned by using the steepest descent until touching a tolerance of 100kJ/mol. And then, the 10 ns MD simulations were carried out with a time step of 1 fs; the corresponding coordinates were stored every 100 fs. The PME algorithm was used to calculate the electrostatic interactions. All simulations were run under the periodic boundary condition with NVT ensemble by using Berensen's coupling algorithm for keeping the temperature at 310 K and pressure at 1atm. All bonds were constrained by using the LINCS algorithm. The GROMACS 4.0 package was utilized to analyze the results.

## Results and Discussion

### 1. Virtual screening and Core hopping

**Figure 2:**
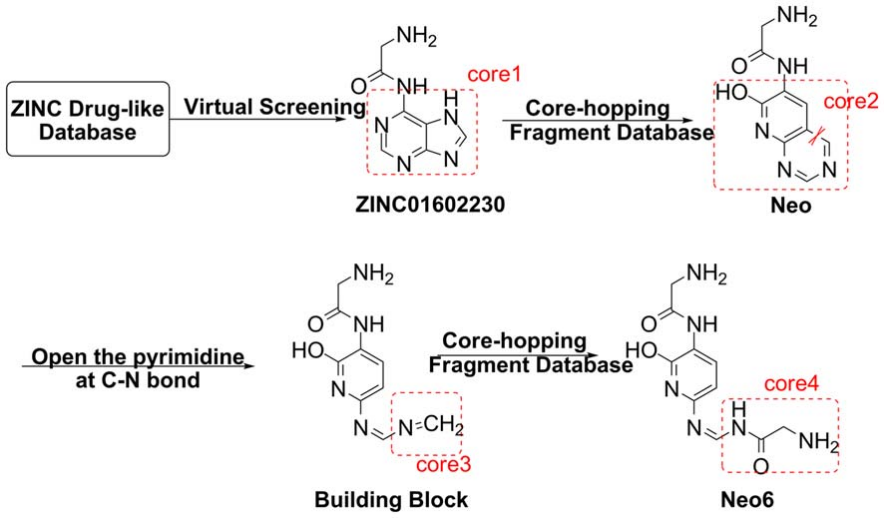
The flowchart to show the inhibitor design process. The core1, core2, core3 and core4 were the key fragments for improving the ligand binding affinity.

The drug-like database from ZINC [38] was screened by using Glide5 for its near-optimal performance aimed on targeting the HA1 receptor (PDB ID:3AL4). The top hit (ZINC01602230) or (2-amino-N-(7H-purin-6-yl) acetamide) (**Fig. 2**), a compound condensation product of Glycine and Adenine, which was considered as the most potential lead compound for further modification. Subsequently, the core hopping method was employed to search the fragment database for replacing the adenine part. Finally, the new structure Neo was discovered that has more strong affinity than ZINC01602230.

The flowchart to show the process of finding the desired inhibitor is given in **Fig. 2**, from which we can see that after the ZINC01602230 was screened out from the Zinc drug-like database, the core hopping method was used to optimize the core1 to core2 by means of searching the ZINC fragment database. The binding affinity between the NAG and the receptor was used as the filtering set. As a result, the compound with the top hits, Neo, was selected for further optimization.

As shown in **Fig. 1**, the rigid core2 fragment in Neo sticking out of the active pocket might not well adhere to the active pocket surface. To improve its binding affinity to the target protein, the bond C-N was cut off at the site shown in **Fig. 2**. As a consequence of doing so, only the pyridine remained and the whole structural flexibility was enhanced so as to have the ability to stretch out to complement the surface of HA1 binding site.

**Figure 3:**
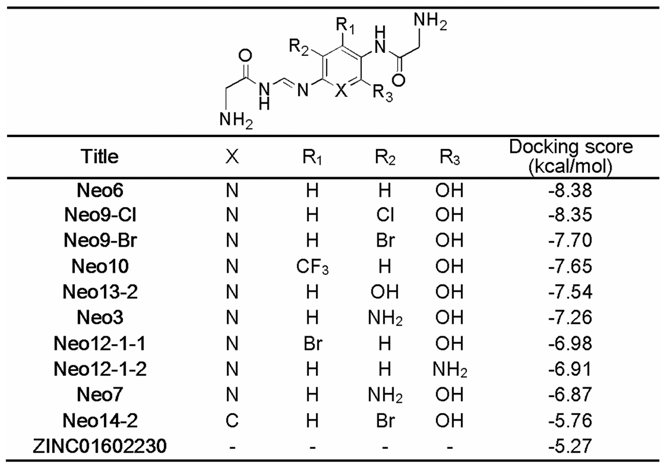
The structures and docking scores list for the top ten compound candidates modified from Neo and ZINC01602230.

The new scaffold as a building block was further optimized through the second core hopping process by replacing the core3 with various fragments by searching the fragment database. Interestingly, the best substitute core4 also contains the same glycine as the terminal fragment on the other side of Neo or ZINC01602230. Subsequently, a series of compound candidates modified from the Neo structure were generated, and then the top ten compounds with the best binding affinity computed by Glide5 program [39], [42] were listed in **Fig. 3**.

### 2. Interactions of the protein with Neo6

**Figure 4:**
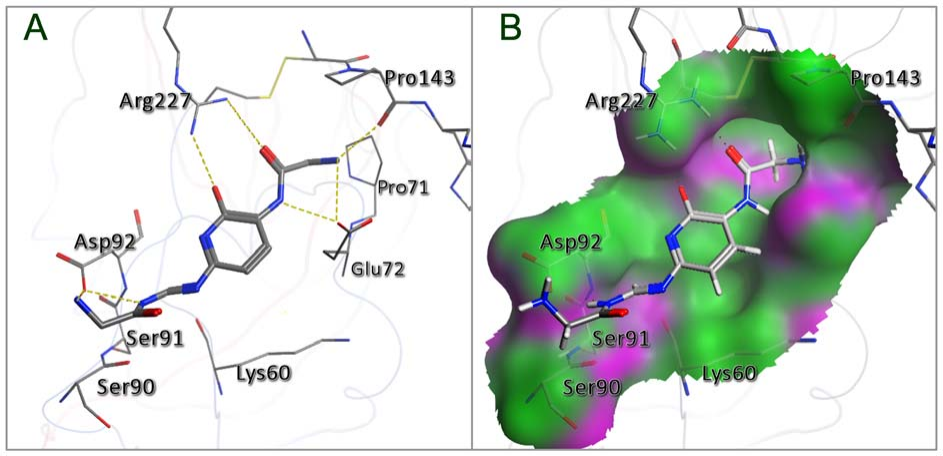
A close view to show the interaction of the receptor with the docked Neo6. (**A**) The yellow dotted lines indicate the H-bond interactions of the receptor with Neo6. (**B**) The molecular surface is shown around the binding site of 3AL4; the hydrophobic surface is colored in green and the hydrogen bond surface in purple. See the text for further explanation.

As can be seen from **Fig. 4**, the result obtained from the docking simulation has proved that the compound binding interactions with residues ARG227 and ASP92 were fully consistent with the previous report [57]. The structure of Neo6 complemented the shallow pocket of HA1 with the optimal conformation. The side chains of the key residues, such as Arg227, Pro143, Glu72 and Asp92 in protein, made a major contribution to the receptor-ligand binding affinity by forming H-bonds with the different heavy atoms (e.g. O, N) of the Neo6 (**Fig. 4**). Besides the common H-bonds formed between the three residues (Arg227, Pro143, and Glu72) and the compound Neo6 as in [58], the other two H-bonds were formed between the two nitrogen atoms of the new extensible fragment core4 and the oxygen atom of Asp92 residue. Consequently, compared with ZINC01602230, the binding affinity of Neo6 with the receptor was strengthened from −5.83 kcal/mol to −8.38 kcal/mol (**Fig. 3**).

### 3. Molecular dynamics trajectory analysis

**Figure 5:**
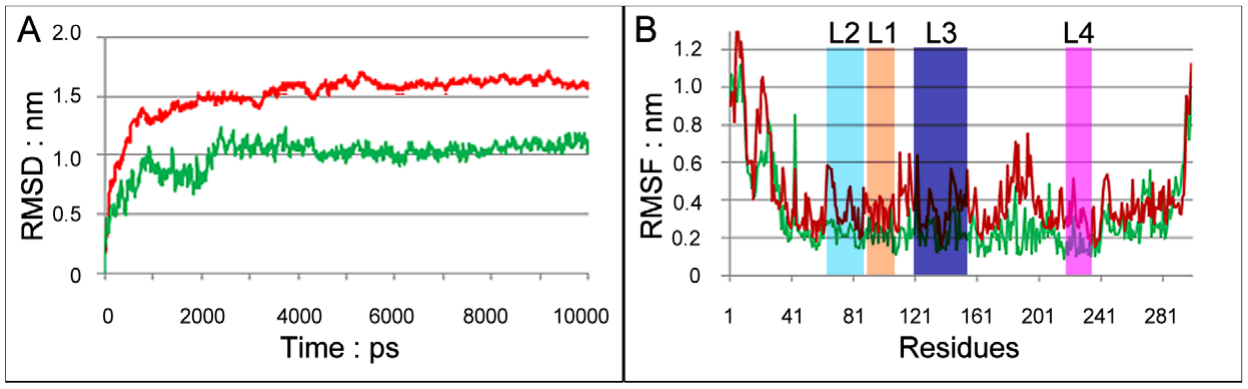
Analysis of molecular dynamics simulations. (**A**) The RMSD for all backbone atoms of the Neo6-HA1 system (green) and the HA1 system (red). (**B**) The RMSF for side-chain atoms of the Neo6-HA1 system (green) and the HA1 system (red). The curves associated with Loop1, Loop2, Loop3, and Loop4 are colored orange, light blue, dark blue, and maroon, respectively.

Furthermore, molecular dynamics simulations were performed for the inhibitor-complexed system HA1-Neo6 and the inhibitor-uncomplexed system HA1, respectively. The root mean square deviation (RMSD) from initial conformation is a central criterion used to evaluate the difference of the protein system. The stability of a simulation system was evaluated based on its RMSD. The RMSD values for both Neo6-HA1 (green curve) and HA1 (red curve) versus the simulation time were illustrated in **Fig. 5A**, in which the RMSD for Neo6-HA1 system is a little smaller than that of HA1 system, indicating that the flexibility of HA1 was decreased after the Neo6 binding to HA1. In order to investigate the motions about the important residues interacted with the inhibitor in the binding site defined as loops (Loop1–Loop4) in **Fig. 1**, the root mean square fluctuations (RMSF) for all the side-chain atoms of protein were calculated, as shown in **Fig. 5B**. The curves of RMSF associated with Loop1, Loop2, Loop3, and Loop4 are colored orange, light blue, dark blue, and maroon, respectively. It can be clearly seen from **Fig. 5** that the fluctuating magnitudes of the four loops in HA1 are much larger than those in Neo6-HA1, clearly indicating that the receptor HA1 is more stable after binding with the ligand Neo6.

Accordingly, among the series of Neo compound candidates, Neo6 is anticipated to be a promising drug candidate for further experimental investigation to develop new and effective drug against influenza viruses.
